# Disruption of redox balance in glutaminolytic triple negative breast cancer by inhibition of glutaminase and glutamate export

**DOI:** 10.1016/j.neo.2025.101136

**Published:** 2025-02-11

**Authors:** Hoon Choi, Mamta Gupta, Arjun Sengupta, Emma E. Furth, Christopher Hensley, Aalim M. Weljie, Hsiaoju Lee, Yu-Ting Lu, Austin Pantel, David Mankoff, Rong Zhou

**Affiliations:** a Department of Radiology, School of Medicine, University of Pennsylvania, Philadelphia, PA, USA; b Department of Systems Pharmacology, School of Medicine, University of Pennsylvania, Philadelphia, PA, USA; c Department of Pathology, School of Medicine, University of Pennsylvania, Philadelphia, PA, USA; d Abramson Cancer Center, University of Pennsylvania, Philadelphia, PA, USA

**Keywords:** Triple-negative breast cancer, Chemo-resistance, Glutaminase, cystine transporter, Redox, Glutathione

## Abstract

Resistance to chemotherapy is an important challenge in the clinical management of triple-negative breast cancer (TNBC). Utilization of the amino acid glutamine as a key nutrient is a metabolic signature of TNBC featuring high glutaminase (GLS) activity and a large pool of cellular glutamate, which mediates intracellular enrichment of cystine via xCT (SLC7A11) antiporter activity. To overcome chemo-resistant TNBC, we identified a strategy of dual metabolic inhibition of GLS and xCT to sensitize resistant TNBC cells to chemotherapy. We successfully tested this strategy in a human TNBC line and its chemoresistant variant *in vitro* and their xenograft models *in vivo*. Key findings of our study include: 1. Dual metabolic inhibition induced pronounced reductions of cellular glutathione accompanying significant increases of cellular superoxide level in both parent and resistant TNBC cells. While GLS and xCT inhibition did not directly kill cells via apoptosis, they potentiated doxorubicin (DOX) and cisplatin (CIS) to induce remarkably higher levels of apoptosis than DOX or CIS alone. 2. Although the resistant TNBC cells exhibited higher capacity to mitigate oxidative stress than the parent cells, their resistance was overcome by dual metabolic inhibition combined with DOX or CIS. 3. In vivo efficacy and safety of the triple combination (GLS and xCT inhibition plus DOX or CIS) were demonstrated in both chemo sensitive and resistant TNBC tumors in mice. In conclusion, GLS and xCT inhibition resulted in unmitigated oxidative stress due to depletion of glutathione, representing a promising strategy to overcome chemoresistance in glutamine-dependent TNBC.

## Highlights

• TNBC cells exhibit high glutaminase activity and maintain a large cellular glutamate pool.
• Blockade of glutaminase and glutamate/cystine antiporter induced oxidative stress.
• Dual blockade plus doxorubicin depleted cellular glutathione and induced apoptosis.
• Dual blockade enhanced cisplatin efficacy to overcome chemoresistant TNBC tumor.
• Changes of metabolites induced by metabolic blockade precede changes in mRNA.

## Introduction

Triple negative breast cancer (TNBC) is an aggressive subtype of breast cancer defined by the absence of estrogen receptors (ER), progesterone receptors (PR), and human epidermal growth factor receptor 2 (HER2). Unlike other subtypes, such as ER/PR positive BC that benefit from endocrine therapies or HER2 positive BC that can be treated by HER2-targeting antibody or antibody-conjugated drugs, TNBC has fewer subtype-specific treatment options. Hence, systemic chemotherapy remains an important component of treatment. Chemotherapy serves as neoadjuvant or adjuvant therapy for TNBC patients with resectable tumors and as a part of first line therapy for metastatic disease. In the setting of immune therapy employing immune checkpoint inhibitor (ICI), ICI is combined with chemotherapy for both early stage and metastatic TNBC[4]. Despite initial responses to chemotherapy, resistance develops frequently, which leads to cancer relapse or progression, highlighting the need for innovative strategies to overcome chemotherapy resistance [5,6].

**Fig. 1:**
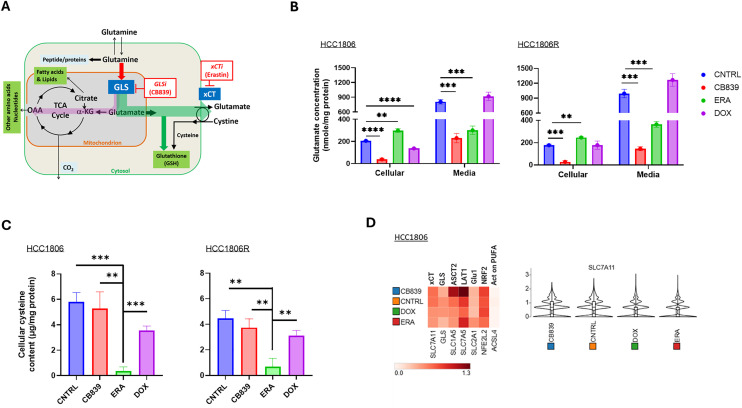
**Role of glutaminolysis and xCT in glutathione synthesis and analyses of cellular metabolites and single cell RNA.** A: The glutaminolysis pathway starts with glutamine transportation into the cell via specific amino acid transporters including ASCT2 and follows the purple arrow. The role of glutamate in *de novo* synthesis of glutathione (GSH) is depicted by green arrows. **B:** Intracellular glutamate and extracellular glutamate concentration after incubation in media (DMEM without glutamate nor FBS) containing CB839 (6 µM), ERA (18 µM), DOX (1.2 µM) or CNTRL (no drug) for 6 hours. **C:** Cellular cysteine concentration. **D:** Single cell RNA sequencing analysis was performed after incubation in media (RPMI1640+10 % FBS) containing CB839 (1 μM), ERA (3 μM), DOX (0.2 μM) or CNTRL (or drug) respectively for 24 hours. Cell number analyzed for scRNAseq: CNTRL=7,377, CB839 =8,768, DOX =6,351 and ERA =9,231, replicate=1. ASCT2 and LAT1 are glutamine transporters and Glu1 glucose transporter. NRF2 is a transcription factor regulating cellular defense of oxidative stress [1,2] while ACSL4 regulates ferroptosis sensitivity in cancer cells [3].

TNBC deploys glutaminolysis pathway (purple arrow in Fig. 1**A**) to utilize glutamine as an essential nutrient in addition to glucose [[7], [8], [9], [10], [11]]. To target this metabolic signature, a highly specific and potent inhibitor of kidney-type glutaminase (GLS), CB839 (Telaglenastat) has been developed [9] that blocks the conversion of glutamine to glutamate, the first and rate-limiting step of glutaminolysis. Clinical trials of CB839 have demonstrated an excellent safety profile, however, anti-cancer efficacy observed has been variable [[12], [13], [14]].

Emerging research has unveiled that glutamate, which is a product of glutaminolysis and also a key component for *de novo* glutathione (GSH) synthesis pathway (green arrows in Fig. 1**A**), may play an important role in maintaining cellular redox homeostasis. Reactive oxygen species (ROS) are produced from physiological processes of cells including OXPHOS in mitochondria, NADPH oxidase activity, growth factor receptor engagement, and exposure to xenobiotics or radiation [15,16]. Due to aberrations in metabolic and growth signal regulation, cancer cells exhibit elevated ROS level than normal cells, hence having a greater demand for cellular antioxidants because unmitigated ROS leads to disruption of cellular redox balance, triggering cell death via apoptosis [17] and/or ferroptosis mediated by lipid peroxidation [[18], [19], [20]]. The GSH synthesis pathway is essential to the initiation of TNBC [21,22], consistent with its role in maintaining redox homeostasis at all stages of cancer evolution including initiation, progression, metastasis, and survival in responses to oxidative treatments [2].

Cellular glutamate contributes to GSH synthesis in two main aspects (green arrows in Fig. 1**A**): first, via antiport activity of xCT (SLC7A11) [23], glutamate facilitates the enrichment of intracellular cystine, a rate limiting substrate for GSH synthesis [24]; second, glutamate is directly incorporated into GSH molecule, a L-γ-glutamyl-L-cysteinyl-glycine tripeptide. Expression of xCT is prevalent in human TNBC [25]. However, xCT inhibition by small molecules such as Erastin or its analogs showed limited efficacy in clinical trials [26]. As demonstrated in a lung cancer model, glutamate utilized for GSH synthesis would limit its availability for the TCA cycle, especially when cellular glutamate pool is reduced by GLS blockade via CB839 [1]. The importance of glutaminolysis and xCT transport in GSH synthesis, with glutamate being the nexus of the two pathways, motivated us to examine a dual metabolic inhibition approach (Fig. 1**A**)*.* In studies presented herein, we tested the hypothesis that pharmacological blockade of xCT and GLS deplete cellular GSH, leading to unbalanced redox state that drives apoptosis and/or ferroptosis in TNBC cells. We explored this approach for overcoming chemotherapy resistant TNBC by *in vitro* and *in vivo* studies. The outcomes of these investigations suggest that this translational strategy holds significant promise in sensitizing resistant TNBC to chemotherapy.

## Materials and Methods

Human TNBC cell line (HCC1806) was purchased from ATCC (catalog CRL-2335). HCC1806R is a paclitaxel resistant line derived by our laboratory from HCC1806 (parent) by exposure to incremental concentration of paclitaxel as we previously described [27]. The cell lines were authenticated using the short-tandem-repeat DNA profiling method. GLS inhibitor (GLSi) CB839 (Calithera Biosciences, Palo Alto, CA) was formulated in dimethyl sulfoxide for cell studies or in vehicle solution as described earlier [9] for in vivo administration. The following chemicals were purchased from Cayman Chemical (Ann Arbor, MI): xCT inhibitor (xCTi) Erastin (ERA) and its analog, Imidazole ketone Erastin (IKE); from ThermoFisher Scientific: Annexin V-FITC, TO-PRO-3, dihydroethidium (DHE, catalog D11347), C11-Bodipy (catalog D3861), and buffer (catalog 00-4222-26) for Fluorescence-activated cell sorting (FACS); from Sigma or Millipore Sigma: Accumax (catalog A7089), glutamate (catalog D5030), doxorubicin (DOX), cisplatin (CIS) and paclitaxel (PTX) in pharmaceutical grade.

### In vitro studies

HCC1806 and HCC1806R cells were cultured in RPMI1640 media (catalog MT10-040-CM, Corning, NY), DMEM (D5030, Sigma-Aldrich, St. Louis, MO) supplemented with 10 % FBS (catalog MT35-010-CV, Corning, NY). No antibiotics were used in the culture.

***Estimation of IC50 of paclitaxel, DOX and cisplatin*** are detailed in Supplemental Information.

***Single cell RNA sequencing (scRNAseq) and analysis*** are detailed in Supplemental Information.

#### Estimation of intra and extracellular glutamate and intracellular cysteine concentrations

One million cells per dish were seeded in 10 cm cell culture dishes and incubated in culture media (DMEM) without glutamate but containing glutamine (0.584 g/l), glucose (1 g/l), and NaHCO3 (3.7 g/l) with 10 % FBS for 24 h at 37 °C. The following day, the cells were treated with CB839 (6 µM), ERA (18 µM), or DOX (1.2 µM) in the same media without FBS for 6 hours followed by collection of the culture media. The cells were then collected by scraping in 1 ml of ice-cold PBS. Both the media and cells were lyophilized using Labconco™ FreeZone™ 4.5L (Fisher Scientific) and kept in -80 °C freezer. To determine the glutamate content in the samples, the lyophilized powder was reconstituted with deionized (DI) water and processed using the Sigma Glutamate Assay Kit (catalog no: MAK330, Sigma Aldrich) while the protein content was measured using the Pierce™ BCA Protein Assay Kit (catalog no: 23225, ThermoFisher Scientific), following manufacturers’ instructions.

To measure cellular cysteine concentration by Liquid Chromatography Mass Spectrometry (LC/MS), 5 million cells/dish were seeded and attached overnight in culture media (RPMI1640+10 % FBS) at 37 °C with 5 % CO2. Subsequently, the media was replaced with that containing CB839 (1 µM), ERA (3 µM), DOX (0.2 µM) and CIS (5 µM) and incubated for 24hrs at 37 °C with 5 % CO2. Afterwards, the cells were washed twice with PBS, and incubated in 1 mL of extraction solution (40 % Methanol, 40 % Acetonitrile, 20 % DI water, 100 mM Formic acid, and 1 mM EDTA) [28]. The extracted cell and solution were collected using a cell scraper and transferred to tubes on ice. For tumor tissue samples, they were homogenized at 4 °C in the same extraction solution (1 mL per 0.1g of tissue) by a Precellys Evolution Homogenizer equipped with Cryolys® Evolution. Extracted samples were centrifuged at 16,000 x g for 10 min, and the supernatant was collected and transferred to tubes on ice. To each tube, 50 μL of internal standard solution (U-^13^C-^15^N-cysteine, catalog no: CNLM-3871-H-PK, Cambridge Isotope Laboratories catalog) was added, and 450 μL of extracts was transferred to corresponding reaction tubes. To each reaction tube, 50 μL of triethylamine was added followed by 5 µL of benzyl chloroformate. The tube was capped, briefly vortexed, and incubated at 37 °C for 10 min. After the incubation, the reaction tubes were centrifuged at 6,000 x g and 4 °C for 5 min. The supernatant was collected for LC/MS analysis of cysteine using a Waters Acquity UPLC system (equipped with a Waters TUV detector at 254 nm and a Waters SQD single quadrupole mass analyzer with electrospray ionization). The LC gradient used was 500 uL/min with a 30 s hold at 95:5 (water: acetonitrile with 0.1 % v/v formic acid), a 2-min gradient to 5:95, and a 30 s hold. An Acquity UPLC HSS C18, 1.7 μm, 2.1 × 50 mm column was employed for the analysis. The data was analyzed using NOVA LC/MS software by Mestrelab Research (https://mestrelab.com/).

#### Estimation of superoxide and lipid peroxidation level (ferroptosis) in cancer cells

HCC1806 and HCC1806R cells were seeded in a 12-well plate and allowed to culture overnight in culture media (RPMI1640+10 % FBS). Subsequently, the media was replaced with culture media containing specified metabolic inhibitor or chemotherapy drug alone and all possible combinations: CB839 (1 µM), ERA (3 µM), DOX (0.2 µM), and the cells were then cultured for an additional 24 hours followed by incubation with 10 µM dihydroethidium (DHE, probe for superoxide) or 2 µM C11-Bodipy (probe for lipid peroxidation / ferroptosis) as final concentration at 37 °C for additional 30 min. For combination treatments involving two or three drugs, the concentrations of each drug remained the same as used for the single-agent treatment. Following three washes with PBS, the cells were detached using Accumax diluted with FACS buffer. Fluorescent intensity of the oxidized DHE and oxidized C11-Bodipy respectively, was measured using FACS (BD Bioscience), and analyzed using FlowJo software. Specified fluorophore (bandpass filter wavelength in nm/width)/cell number were as follows: oxidized C11-Bodipy (530/30 nm)/10,000 cells and oxidized DHE (610/20 nm)/10,000 cells.

#### Estimation of early apoptosis by FACS/cell sorting

Cell culture and treatment were the same as described in Estimation of superoxide. After incubation, the cells were collected and washed 3 times with PBS. The collected cells were resuspended in 100 μL of HEPES buffer solution (10 mM HEPES, pH 7.4). Annexin V-FITC (5 μL) for staining early apoptosis and TO-PRO-3 (20 μL) for dead cells were added to the cell suspension and incubated at room temperature for 15 min followed by FACS on a BD LSRII flow cytometer (BD Bioscience), with fluorophore (bandpass filter wavelength in nm/width)/cell number as described: Annexin V-FITC (530/30 nm)/10,000 cells, and TO-PRO-3 (660/20 nm)/10,000 cells. The FACS data were analyzed by FlowJo software (BD Bioscience) to determine the extent of early apoptosis.

#### Estimation of cellular glutathione (GSH) level

200K cells were seeded per well in 12-well plate. Cell culture and treatment were the same as described in Estimation of superoxide. After incubation, the cells were detached using trypsin and centrifuged at 700 x g for 5 min at 4 °C. The supernatant was removed, the cell pellet was resuspended in 0.5 mL of ice-cold PBS, transferred to a 1.5 mL microcentrifuge tube and centrifuged at 700 x g for 5 min at 4 °C. The supernatant was removed, and the cells were lysed in 80 μL of ice-cold buffer from Glutathione Assay Kit (catalog no: ab239709, Abcam). The lysed cells were kept on ice for 10 min followed by mixing thoroughly with 20 μL of 5 % sulfosalicylic acid and centrifuged at 8000 x g for 10 min. The resulting supernatant was transferred to a fresh tube and GSH content was estimated following the Kit's instructions.

### In vivo studies

All animal procedures were approved by the institutional animal care and usage committee (IACUC) of the University of Pennsylvania.

#### TNBC xenograft models, treatment regimens and tumor growth measurement

To establish the human breast cancer xenografts, one million HCC1806 or HCC1806R cells in 100 μL PBS, were inoculated subcutaneously into the right flank of athymic nu/nu mice (female 7-week-old, Charles River). Tumor size was measured by caliber in two orthogonal directions *a* and *b* with *b* being the shorter dimension using formula: V= πab^2^/6.

For detection of cell death by diffusion-weighted MRI, mice bearing HCC1808 xenografts were enrolled randomly into Control (no treatment) or combination treatment (CB839+ERA+DOX) groups when tumor size reached ∼200 mm^3^ with dose regimens as described below in growth delay study.

For tumor growth delay study of resistant model, mice bearing HCC1808R xenografts were enrolled when the tumor size reached 166 ± 84 mm^3^, and were randomly assigned to one of the 8 groups for 14-day treatment: Control (untreated); CB839 (200 mg/kg administered orally twice daily at Day 0, 1, 2, 3, 4, 7, 8, 9, 10, 11, and 14); ERA (5 mg/kg administered intraperitoneally at Day 0, 2, 4, 7, 9, 11, and 14); CIS (2.5 mg/kg i.p. at Day 0, 2, 4, 7, 9, 11, and 14); and all possible combinations of these three drugs: CB839+ERA, CB839+CIS, ERA+CIS and CB839+ERA+CIS. For the combinations, the dose regime and injection route were the same as those used for the single-drug treatment. Tumor size was measured every other day by caliber. The mouse was sacrificed when the tumor reached the size of 1000 mm^3^ or after being treated for two weeks, whichever occurred earlier, and the tumor was harvested for further analysis.

#### Diffusion-weighted MRI and data analyses

MRI studies were performed on a 9.4 T Avance III console (Bruker, Berillica, MA, USA), equipped with 12 cm ID, 40 G/cm gradients. Details of diffusion-weighted image acquisition, reconstruction and analyses to derive ADC map of the tumor were described in our prior study [29]. After MRI, the mouse was euthanized, and tumor tissue harvested with portions fixed in formalin or clamp-frozen in liqN2, respectively.

### Ex vivo studies

#### Estimation of tissue glutamine and glutamate concentration by ^1^H NMR

Tumor was clamp-frozen in LiqN2 and kept in -80 °C freezer. Metabolites were extracted using a modified Bligh-Dyer protocol (https://currentprotocols.onlinelibrary.wiley.com/doi/abs/10.1002/cpps.98). Briefly, the frozen tissue (50 mg) was suspended in 200 µL of methanol+chloroform and 100 µL of water precooled to 4 °C. The ice-cold stainless-steel beads were then added to the samples and homogenized at 25Hz for 2.00 min. The homogenized suspension was centrifuged at 13,300 rpm for 10 min at 4 °C. The supernatant (methanol-water) portion was separated and transferred to labeled Eppendorf tubes. The supernatant was freeze-dried using FreeZone4.5 lyophilizer (Labconco Co., Kansas City, MO, USA). Lyophilized powder was resuspended in 200 µL of phosphate buffer (pH ∼ 7) containing 0.25 mM DSS (Sodium trimethylsilylpropanesulfate, Cambridge Isotope Laboratories, Andover, MA) and 10 % deuterium oxide and transferred to a 3 mm NMR tube.

One-dimensional ^1^H NMR spectra were acquired at 298°K on a Bruker AVANCE-III HD 700 MHz spectrometer (Bruker Biospin, Billerica, MA) fitted with a 3 mm TXI probe. The pulse program took the shape of first transient of a 2 dimensional NOESY and generally in the form RD-90-t-90-tm-90-ACQ. Where RD = relaxation delay, t = small time delay between pulses, tm = mixing time and ACQ = acquisition. The water signal was saturated using continuous irradiation during RD and tm. The spectra were acquired using 76K data points and 14 ppm spectral width. 1024 scans were performed and 1 s interscan (relaxation) delay and 0.1 s mixing time was allowed. The FIDs were zero filled to 128K; 0.1 Hz of linear broadening was applied followed by Fourier transformation. NMR spectra were imported into Chenomx v 8.0. (Edmonton, Canada) for quantitative targeted profiling [30]. The processor module was used to correct phase and baseline of the spectra followed by internal standard calibration and deletion of water region. The processed spectra were then imported to the profiler module for targeted profiling. Metabolites concentration was normalized to the tissue wet weight.

#### Immunohistochemistry of tumor tissues

Immunohistochemistry (IHC) of apoptosis was applied on FFPE (formalin fixed paraffin embedded) sections using anti-casepase-3 antibody by the Pathology Core of Children's Hospital of Philadelphia. IHC protocols are posted on the Core's website (https://www.research.chop.edu/pathology/tools, accessed on 16 August 2022). Stained sections were scanned at × 40 magnification using Aperio ScanScope CS2 (Leica Biosystems Imaging, CA, USA), and digital pictures were uploaded to QuPath4.0 for analyses [31] by a pathologist (EEF) with over 20 years’ clinical practice.

### Statistical Analysis

Data were presented as mean ± standard deviation with sample size specified in figure captions. Statistical analyses were performed in Prism GraphPad (San Diego, CA) with the level of α set at 0.05 for evaluation of statistical significance.

## Results

A paclitaxel-sensitive and -resistant human TNBC line, HCC1806 and HCC1806R, respectively were used to evaluate the dual metabolic blockade approach. The HCC1806R exhibits 35-, 4- and 3-fold increase in IC50 to paclitaxel (PTX), doxorubicin (DOX), and cisplatin (CIS), respectively compared to the parent line (**SI** Fig. 1). We first examined intra- and extracellular glutamate after exposure to GLSi, xCTi or DOX for 6 hours and cellular cysteine after exposure for 24 hours. Our data show that CB839 reduced intra- and extracellular glutamate concentration significantly in both parent and resistant cells (Fig. 1**B**), consistent with reduced glutamate production upon GLS inhibition. By blocking xCT antiporter that mediates glutamate export and cystine import (green arrows in Fig. 1**A**), ERA reduced glutamate concentration in the media and dramatically decreased cellular cysteine concentration (Fig. 1**C**), Exposure to DOX did not affect extracellular glutamate or cysteine level in resistant or parent cells (Fig. 1**B, C**). These data suggest that GLS and xCT blockade led to swift declines in cellular glutamate and cysteine, both of which are required for GSH synthesis. In contrast, 24-h exposure did not alter mRNA level of GLS or xCT (SLC7A11) based on single cell RNA sequencing analysis (Fig. 1**D**), suggesting that metabolite changes preceed the change in GLS or xCT gene expression levels.

**Fig. 2:**
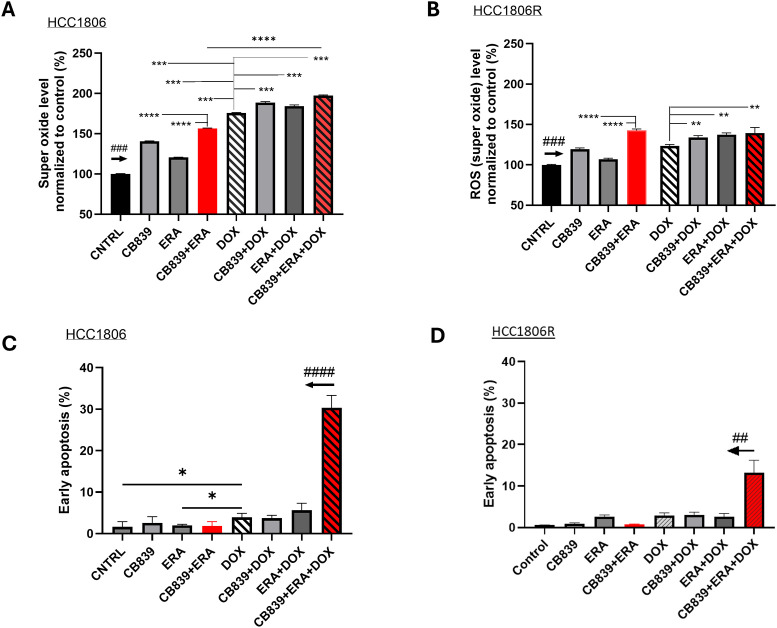
**Impact of metabolic blockade and/or doxorubicin on cellular superoxide level and apoptosis in chemo sensitive and resistant TNBC cells. A-B:** Super oxide level (normalized to untreated controls set at 100) in cells exposed to CB839, ERA, DOX and all possible combinations, respectively (N=4 replicates for each treatment). The arrow above CNTRL indicates ### *P* < 0.001 comparing CNTRL vs. other treatments in the panel. **C-D:** Early apoptosis estimated by FACS (N=3 replicates for each bar). The arrow above CB839+ERA+DOX indicates #### *P* < 0.0001 (HCC1806) or ## *P* < 0.01 (HCC1806R) comparing the combination treatment vs. other treatments in the panel.

Next, we examined the impact of GLSi, xCTi, DOX chemotherapy and their combinations to induce oxidative stress associated with increased cellular superoxide and lipid peroxidation level. We are particularly interested in dual metabolic inhibition (GLSi + xCTi) and its combination with chemotherapy. As shown in Fig. 2**A-B**, CB839, ERA, DOX alone or their possible combinations mediated a significant increase of the cellular superoxide in both parent and resistant cells (*P* < 0.001 compared to CNTRL). Dual metabolic inhibition (CB839+ERA) is substantially more effective than single agent in increasing superoxide levels, and the triple combination (CB839+ERA+DOX) led to significantly higher ROS than DOX alone in both parent and resistant cells. Notably, the resistant cells exhibit significantly lower superoxide levels than parent cells across all treatments (**SI** Fig. 2**A**), indicating that resistant cells have developed adaptive mechanism to mitigate oxidative stress above the capacity of the parent cells.

To test whether the increased cellular ROS level directly mediates apoptosis, we estimated the fraction of cells undergoing early apoptosis by FACS after staining cells with Annexin-V (early apoptosis probe) and TO-PRO-3 (**SI** Fig. 3). While DOX alone increased the fraction of apoptotic cells significantly in parent cells (*P* < 0.05), the triple combination (CB839+ERA+DOX) dramatically increased apoptosis compared to all other treatments in parent cells (*P* < 0.0001, Fig. 2**C**), suggesting that, while dual metabolic inhibition in itself does not induce apoptosis, this approach sensitizes the cells to DOX-mediated cell killing. Notably, the resistant cells showed increased apoptosis only with the triple combination (*P* < 0.01, Fig. 2**D**).

**Fig. 3:**
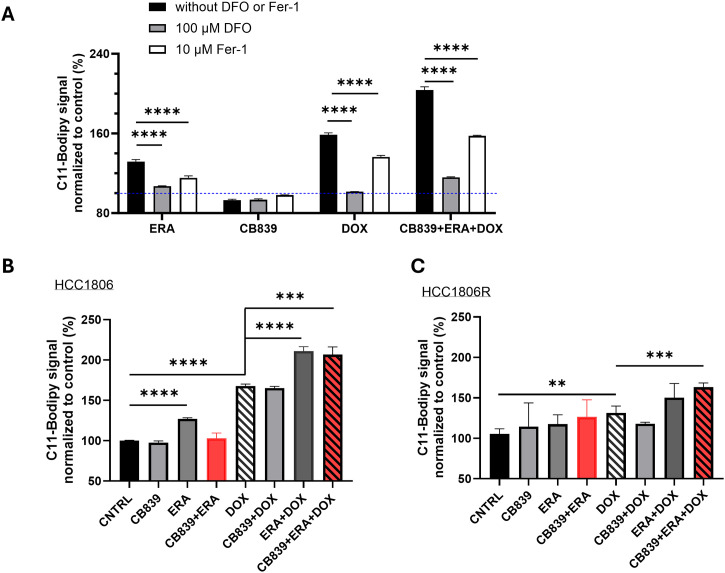
**Impact of metabolic blockade and/or doxorubicin on ferroptosis in chemo sensitive and resistant TNBC cells. A:** C11-BODIPY signal was measured in the presence or absence of DFO, or Fer-1 to confirm the nature of C11-BODIPY signal (normalized to CNTRL set at 100 by dotted blue line). **B, C:** C11-BODIPY signal in HCC1806 and HCC1806R cells after 24 h exposure to CB839, ERA, DOX or their combinations. **P < 0.01, ***P < 0.001, ****P < 0.0001 (N=4 replicates in each group).

For ferroptosis measurements, HCC1806 or HCC1806R cells were incubated with CB839 (1 μM), ERA (3 μM), Dox (0.2 μM) and their combinations, respectively.

Besides inducing apoptosis, cellular ROS causes lipid peroxidation, an irreversible process, activating iron-dependent ferroptosis pathway, which leads to a form of cell death (ferroptosis) independent of apoptosis [18]. To confirm the nature of C11-BODIPY signal as a marker of ferroptosis [20], our data show that adding iron chelator deferoxamine (DFO) or Ferrostatin-1 (Fer-1), a known inhibitor of ferroptosis, to the culture media significantly reduced C11-BODPY signal (Fig. 3**A**). CB839 did not induce ferroptosis over CNTRL (set at 100, dotted blue line) whereas ERA induced a robust C11-BODIPY signal as did DOX. The combination of ERA and DOX led to the largest increase of C11-BODIPY signal (Fig. 3**B**). As it is known to induce lipid peroxidation, DOX alone induced significant ferroptotic cell death in parent and resistant cells compared to CNTRL; however, adding xCT blocker ERA substantially increased ferroptosis compared to DOX alone in parent cells (*P* < 0.001, Fig. 3**B**) whereas in resistance cells, the triple combination was necessary to increase the C11-Bodipy signal over DOX alone (*P* < 0.001, Fig. 3**C**). Again, significantly higher level of ferroptosis was observed in parent cells than resistant cells induced by CB839+ERA as well as the triple combination (**SI** Fig. 2**B**), confirming the enhanced capacity of resistant cells to mitigate lipid peroxidation hence limiting ferroptotic cell death.

**Fig. 4:**
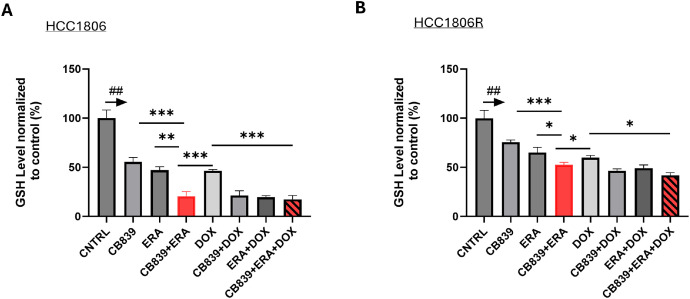
**Impact of metabolic blockade and/or doxorubicin on cellular GSH level in chemo sensitive and resistant TNBC cells.** GSH level is normalized to CNTRL (set at 100) in HCC1806 (**A**) and HCC1806R (**B**) cells after 24 h exposure to specified treatment (triplicate samples for each treatment). The arrows above the CNTRL indicate ## *P* < 0.001 comparing CNTRL vs. any other treatment in the panel. HCC1806 or HCC1806R cells were incubated with CB839 (1 μM), ERA (3 μM), Dox (0.2 μM) or their combinations, respectively for 24 h. *P < 0.05, **P < 0.01, ***P < 0.001.

As the primary cellular antioxidant, GSH mitigates ROS-mediated apoptosis and ferroptosis [19,32]. Our data show that all treatments reduced GSH significantly compared to CNTRL in both parent and resistant cells (*P* < 0.01, Fig. 4**A,B**). Dual metabolic inhibition as well as the triple combination led to the lowest GSH level in both parent and resistant cells, consistent with the ability of these treatments to induce the highest level of apoptosis and ferroptosis compared to other treatments. Again, resistant cells maintained significantly higher levels of GSH than parent cells across all treatments (**SI** Fig. 2**C**).

**Fig. 5:**
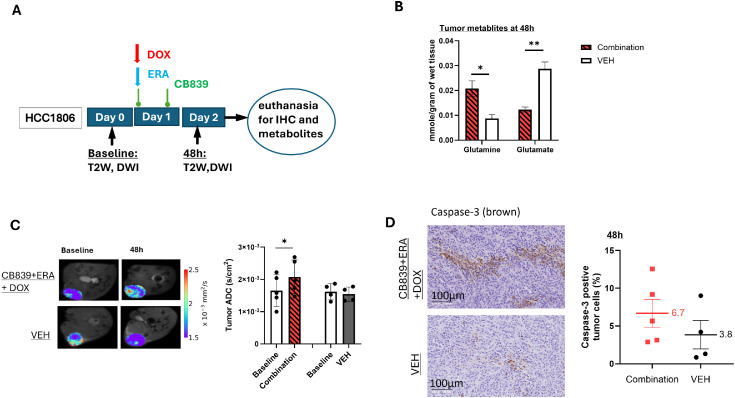
**In vivo and ex vivo studies of HCC1806 xenografts after a short course treatment with triple combination (CB839+ERA+DOX). A:** Study design. **B:** Tumor glutamine and glutamate concentration (mmole/g of wet weight) by ex vivo ^1^H MR spectroscopy. **C:** Detection of cell death by ADC derived from diffusion weighted MRI. **D:** Apoptosis in tumor sections measured by Caspase-3 staining (CC3). *P < 0.05, **P < 0.01 (N= 4 and 5 mice enrolled in CNTRL and treatment group, respectively).

Based on the promising *in vitro* results of the combination treatment (CB839+ERA+DOX), we tested the *in vivo* efficacy of this treatment in the HCC1806 xenograft model by measuring cell death by diffusion weighted MRI (DWI) and Caspase-3 staining. The short treatment course of two days (Fig. 5**A**) is based on our prior studies that GLS inhibition by CB839 were detected at 48h by [^18^F]fluoroglutamine PET or CEST MRI and confirmed by enzymatic assay [33,34]. Consistent with GLS blockade, metabolites analyses revealed significantly reduced glutamate and increased glutamine level in the tumors after the combination treatment compared to CNTRL (Fig. 5**B**). An increase in apparent diffusion coefficient (ADC) of the tumor is considered as a marker of cell death in response to radiation and/or chemotherapies [35]. Our data demonstrated significantly increased ADC values at 48h after the combination treatment compared to baseline in all tumors (*P* < 0.05) whereas tumors in CNTRL mice exhibit no change in ADC (Fig. 5**C**). ADC values are corroborated with a higher level of apoptosis in tumors after combined treatment than CNTRL assessed by Caspase-3 staining however statistical significance was not reached (6.7 % vs. 3.8 %, *P* > 0.05, Fig. 5**D**).

**Fig. 6:**
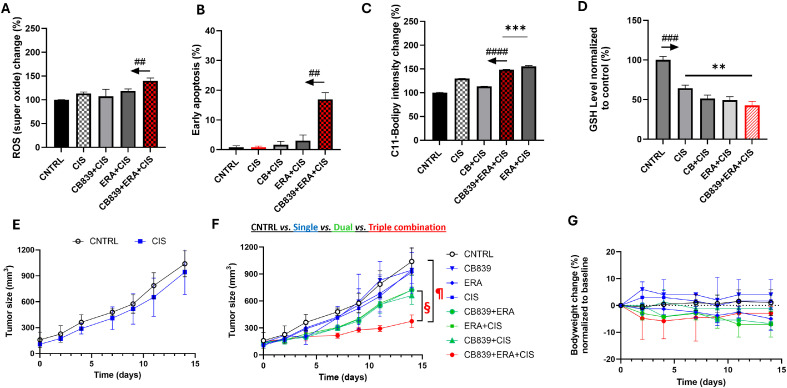
**Dual metabolic inhibition sensitizes chemo-resistant TNBC cells and tumors to cisplatin chemotherapy.** Superoxide (**A**), apoptosis (**B**), ferroptosis (**C**) and GSH (**D**) level in HCC1806R cells after incubation. **E:** CIS treatment did not induce growth delay of HCC1806R tumors. **F:** HCC1806R tumor growth time course during 2-week treatment by CB839 (200 mg/kg twice daily oral), ERA (5mg/kg, ip, 3 times /wk), CIS (2.5 mg/kg, ip, 3 times /wk) and the triple combination, respectively. ^¶^*P* = 0.002, 0.021, 0.016 and 0.003 comparing the triple combination *vs*. CNTRL, CIS, CB839 and ERA, respectively. ^§^*P* = 0.026, 0.002, 0.003, comparing the triple combination vs. CB839+ERA, CB839+CIS, and ERA+CIS, respectively; *P < 0.05 comparing CB839+CIS, ERA+CIS respectively vs. CNTRL. **G:** %change of bodyweight of mice during treatment in E.

We then tested the extent to which dual blockade enhances the efficacy of other forms of chemotherapy used to treat TNBC besides DOX. We studied both paclitaxel and cisplatin (**SI** Fig. 1), and we chose CIS as a treatment that is independent of the drug (paclitaxel) used to generate the resistant cell line. We therefore examined the *in vitro* and *in vivo* effects of dual metabolic inhibition combined with CIS on resistant cells and tumors. Compared to all other treatments, the combination treatment (CB839+ERA+CIS) induced a significantly higher level of super oxide (*P* < 0.01, Fig. 6**A**) and apoptosis (*P* < 0.01, Fig. 6**B**). Furthermore, the triple combination led to significantly higher ferroptosis (C11-BODYPI) signal than CIS alone or CIS+CB839 (*P* < 0.0001, Fig. 6**C**), whereas ERA+CIS induced higher C11-BODYPI signal than the triple combination (*P*< 0.001), consistent with Fig. 3**B** that CB839 did not enhance but rather mitigate ERA-induced lipid peroxidation and ferroptosis despite its contribution to superoxide and induction of apoptosis. These results mirror those of DOX-based combination on resistant cells (Fig. 2**B, D** and Fig. 3**C**). Treatment with CIS, GLSi and xCTi alone, respectively as well as their dual and triple combinations reduced cellular GSH significantly compared to CNTRL in resistant cells (*P* < 0.001, Fig. 6**D** and Fig. 4**B**).

For super oxide and ferroptosis measurements, HCC1806R cells were incubated with CB839 (1 μM), ERA (3 μM), CIS (5 μM). For early apoptosis measurement, HCC1806R cells were incubated with ERA (6 μM), CB839 (2 μM), CIS (10 μM) and their combinations, respectively for 24 h.

In xenograft models, CIS retarded the growth of HCC1806 tumors (**SI** Fig. 4) but failed to do so in HCC1806R tumors (Fig. 6**E**) as we expected. CB839 or ERA alone did not impact the tumor growth either (blue symbols in Fig. 6**F**) nor did their combination (green symbol, *P* = 0.065) compared to the CNTRL, consistent with findings for ferroptosis and apoptosis levels in the cell studies (Fig. 2**D** and 3**C**). While CIS combined with CB839 or with ERA delayed the tumor growth moderately compared to the CNTRL (green symbols, *P* <0.05, Fig. 6**F**), the triple combination (CB839+ERA+CIS) was able to overcome resistance to CIS, inducing a significant tumor growth delay (red symbol) comparing to treatments by CIS alone or two-drug combinations (¶ and §, Fig. 6**F,** *P* values specified in the caption). Promisingly, the triple combination treatment was well tolerated with less than 10 % body weight loss and is not significant compared to body weight at baseline (Fig. 6**G**, and **SI** Fig. 5 for the actual body weight over time**)**. In summary, in CIS-resistant tumor, dual metabolic inhibition sensitizes the cancer cells, leading to significant tumor growth delay after CIS-based combination treatment.

## Discussions

Enhanced oxidative stress in rapidly dividing cancer cells is an important factor mediating the efficacy of many cytotoxic chemotherapy agents [[36], [37], [38], [39]]. Our study revealed that chemo-resistant TNBC cells relying on glutamine metabolism were able to maintain lower levels of cellular superoxide and lipid peroxidation in the face of chemotherapy compared to their chemo-sensitive counterpart (**SI** Fig. 2**A, B**), providing a plausible mechanism for resistance and a motivation for disrupting redox balance as a strategy to abrogate chemotherapy resistance. Our prior work using both radioisotope and stable isotope metabolic tracing of glutamine revealed that glutamine metabolism through GLS contributed to a large pool of cellular glutamate [40,41] that can provide both glutamate (directly) and cysteine (indirectly via xCT) required for de novo synthesis of GSH (Fig. 1**A**). Leveraging this insight, we investigated the potential of targeting glutamate production via GLS and export via xCT using dual metabolic blockade to disrupt cellular redox balance and sensitize resistant TNBC tumors to chemotherapy.

Our results reveled significant changes in cellular glutamine, glutamate and cysteine upon exposure to GLSi (CB839) and xCTi (ERA) and metabolite changes precede the transcriptome changes of targeted proteins (Fig. 1**B-D**). These data suggest that imaging methods capable of assessing cellular glutamate, glutamine and cysteine concentration can be employed to assess pharmacodynamic effect of GLS and xCT inhibitor, respectively. These methods include GluCEST MRI, [^18^F]fluciclovine, [^18^F](2S,4R)4-Fluoroglutamine ([^18^F]-4F-Gln) and (4S)-4-(3-[^18^F]fluoropropyl)-l-glutamate ([^18^F]FSPG) PET that we and other investigators have studied [33,34,[42], [43], [44], [45], [46]].

Dual metabolic blockade increased cellular superoxide level remarkably (Fig. 2) and led to depletion of cellular GSH (Fig. 4, Fig. 6) as expected since the combination of GLS and xCT antagonist reduced the availability of key molecules needed for GSH synthesis. Dual metabolic blockade, when combined with DOX, led to highly significant increases in apoptosis *in vitro* (Fig. 2**C**) and *in vivo* in HCC1806 tumors (Fig. 5**C-D**), likely mediated by disrupted cellular redox balance due to depletion of GSH. Furthermore, DOX's ability to induce lipid peroxidation-meidated cell death (ferroptosis) was bolstered significantly by addition of ERA (Fig. 3**B**). This implies a potentially broader impact on chemotherapy efficacy beyond DOX, and in fact, we found that dual blockade significantly increased CIS efficacy in treating resistant TNBC (HCC1806R) both in vitro and in vivo (Fig. 6). GSH depletion is a plausible mechanism for enhancing CIS cytotoxicity, since it prevents the formation of CIS-GSH conjugates, which are exported out of cells thereby allowing cancer cells to evade CIS-mediated DNA damage and cell death [47]. These data support a broader mechanism of overcoming chemotherapy resistance by depleting GSH in cancers that are dependent upon glutamine metabolism. Overall, our data supports GSH depletion as mechanism underpinning the sensitization of resistant TNBC to CIS chemotherapy consistent with our hypothesis that motivated dual metabolic blockade (Fig. 1**A**).

Our data sheds an insight into the lack of cytotoxic effect of CB839 as single agent [9]. Although it robustly increased cellular super oxide level (Fig. 2**A**) that consequently diminished cellular GSH pool by 50 % (Fig. 4**A**), CB839 alone had no impact on inducing apoptotic (Fig. 2**C**) or ferroptotic cell death (Fig. 3**B**). In vivo, CB839 or ERA did not enhance CIS chemotherapy in resistant tumors (*P* > 0.05 comparing CIS vs. CB839+CIS or ERA+CIS, Fig. 6**F**) despite the data that CIS plus CB839 or ERA induced a moderate growth delay compared to the CNTRL. In contrast, the triple combination (CB839+ ERA+ CIS) overcame CIS-resistance by mediating a significant growth delay in HCC1806R tumors (*P* < 0.05, comparing CB839+ERA+CIS vs. CIS**,** Fig. 6**F**).

In summary, our study provides compelling evidence for the therapeutic benefit and feasibility of dual metabolic blockade as a translational strategy to sensitize resistant TNBC to cytotoxic chemotherapy, suggesting a rationale for further mechanistic studies and additional test that could leverage the precision imaging methods developed to monitor the pharmacodynamic effect of glutaminase inhibitor and xCT activity *in vivo* [33,34,[42], [43], [44], [45], [46]].

## CRediT authorship contribution statement

**Hoon Choi:** Conceptualization, Data curation, Formal analysis, Investigation, Methodology, Validation, Writing – original draft, Writing – review & editing. **Mamta Gupta:** Data curation, Formal analysis. **Arjun Sengupta:** Data curation, Methodology. **Emma E. Furth:** Data curation, Methodology, Writing – review & editing. **Christopher Hensley:** Data curation, Methodology, Writing – review & editing. **Aalim M. Weljie:** Data curation. **Hsiaoju Lee:** Data curation, Resources. **Yu-Ting Lu:** Data curation. **Austin Pantel:** Conceptualization. **David Mankoff:** Conceptualization, Funding acquisition, Investigation, Resources, Supervision, Validation, Writing – original draft, Writing – review & editing. **Rong Zhou:** Conceptualization, Data curation, Formal analysis, Funding acquisition, Investigation, Methodology, Resources, Supervision, Validation, Writing – original draft, Writing – review & editing.

## Declaration of competing interest

The authors declare that they have no known competing financial interests or personal relationships that could have appeared to influence the work reported in this paper.

## Appendix Supplementary materials

Image, application 1
