# Supplementary material for: Disruption of redox balance in glutaminolytic triple negative breast cancer by inhibition of glutaminase and glutamate export
Source: Neoplasia. 2025 Feb 11;61:101136. doi: 10.1016/j.neo.2025.101136 (PMC11869985; doi:10.1016/j.neo.2025.101136)
Supplement: Supplementary file 1 [file mmc1.docx]

**Supplementary Information**

**Disruption of redox balance in glutaminolytic triple negative breast cancer by inhibition of glutaminase and glutamate export**

Hoon Choi^1^, Mamta Gupta^1^, Arjun Sengupta^2^, Emma E. Furth^3,4^, Christopher Hensley^1^, Aalim M. Weljie^2^, Hsiaoju Lee^1^, Yu-Ting Lu^1^, Austin Pantel^1^, David Mankoff^1,4^, and Rong Zhou^1,4*^

^1^Department of Radiology, ^2^Department of Systems Pharmacology, and ^3^Department of Pathology, School of Medicine, University of Pennsylvania, Philadelphia, PA, USA, ^4^Abramson Cancer Center, University of Pennsylvania, Philadelphia, PA, USA

*Corresponding to Rong Zhou, PhD, rongzhou@pennmedicine.upenn.edu

**Supplemental Methods**

***Estimation of IC50 of paclitaxel, DOX and cisplatin***

To determine the 50% inhibitory concentration (IC50) of PTX, DOX and CIS, HCC1806 and HCC1806R cells respectively were seeded at 6K cells per well in a 96-well plate and cultured in RPMI1640 supplemented with 10% FBS for 24 hours at 37°C with 5% CO2. Following the initial culture period, the media was replaced with fresh media containing one chemo drug with a range of concentration: PTX (0 to 500 nM), DOX (0 to 50 μM), or CIS (0 to 100 μM), followed by incubation for an additional 72 hours; for each drug, duplicate wells were used for each concentration. To assess cell viability, the cells were washed twice with PBS and subsequently incubated for 2 hours with fresh culture media containing 10 μL of CCK-8 solution (catalog no: NC9261855, Fisher Scientific) added in each well. The percentage of viable cells at each concentration was estimated by the absorbance at 450 nm using a Microplate Reader (SpectraMax M5, Molecular Devices). IC50 value for each chemo drug was determined using GraphPad Prism (version 10.0.2).

***Single cell RNA sequencing (scRNAseq) and analysis***

HCC1806 **Cells were cultured in RPMI1640 medium with 10% FBS containing CB839 (1µM), ERA (3µM), or DOX (0.2 µM) for 24 hours. Afterwards, cells were collected by trypsinization and washed 2 times with PBS. Cells were suspended in 0.04%PBS** **with** concentration ≥ 200K cells/100μL for submission to the Genomic and Sequencing Core at the University of Pennsylvania. Next-generation sequencing libraries were prepared using the 10x Genomics Chromium Single Cell 3’ Reagent kit v3 per manufacturer’s instructions. Libraries are uniquely indexed using the Chromium dual Index Kit, pooled, and sequenced on an Illumina NovaSeq 6000 sequencer in a paired-end, dual indexing run. Sequencing for each library was targeted at 20,000 reads per cell. Data is then processed using the Cell Ranger pipeline (10x Genomics, v.6.1.2) for demultiplexing and alignment of sequencing reads to the mm10 transcriptome and creation of feature-barcode matrices.

To assess the mRNA levels of xCT (SLC7A11), GLS, ASCT2, LAT1, Glu1, NRF2 and ACSL4, we used the Cell Ranger (10X Genomics) output of filtered matrices supplied by the sequencing core to integrate the data for each sample using the Seurat package (v4), followed by normalization and unsupervised clustering by SCTransform (v2). In Bioconductor (v3.16), the SingleR package combined with the celldex databases was used to perform automated cell-type assignments. Cirrocumulus was then employed for interactive exploration and visualization of datasets. Heatmaps and violin plots were generated using Cirrocumulus. Target genes were extracted from literature (1-4).

**Supplemental Figures**

**
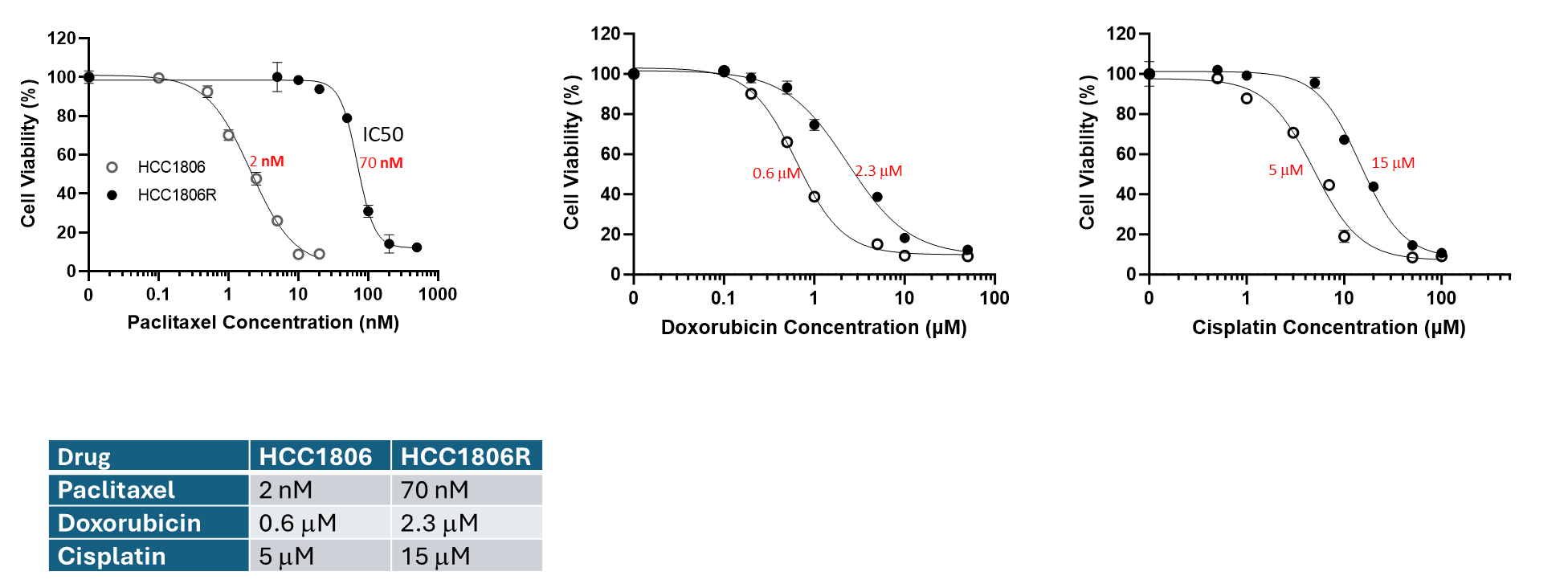
**

**SI Figure 1. Comparisons of IC50 of chemotherapy drugs (paclitaxel, doxorubicin and cisplatin) in parent and resistant human TNBC (HCC1806) cells.**

**
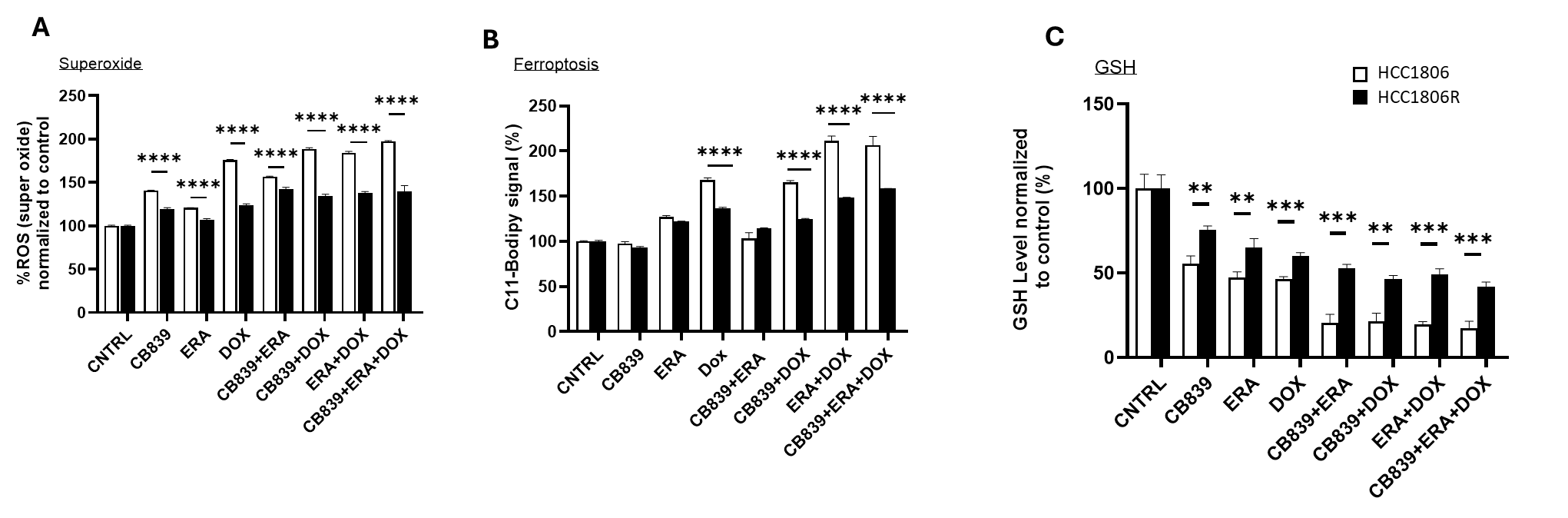
**

**SI Figure 2. Comparisons of superoxide, ferroptosis and GSH level in chemo sensitive and resistant TNBC(HCC1806) cells.**

**
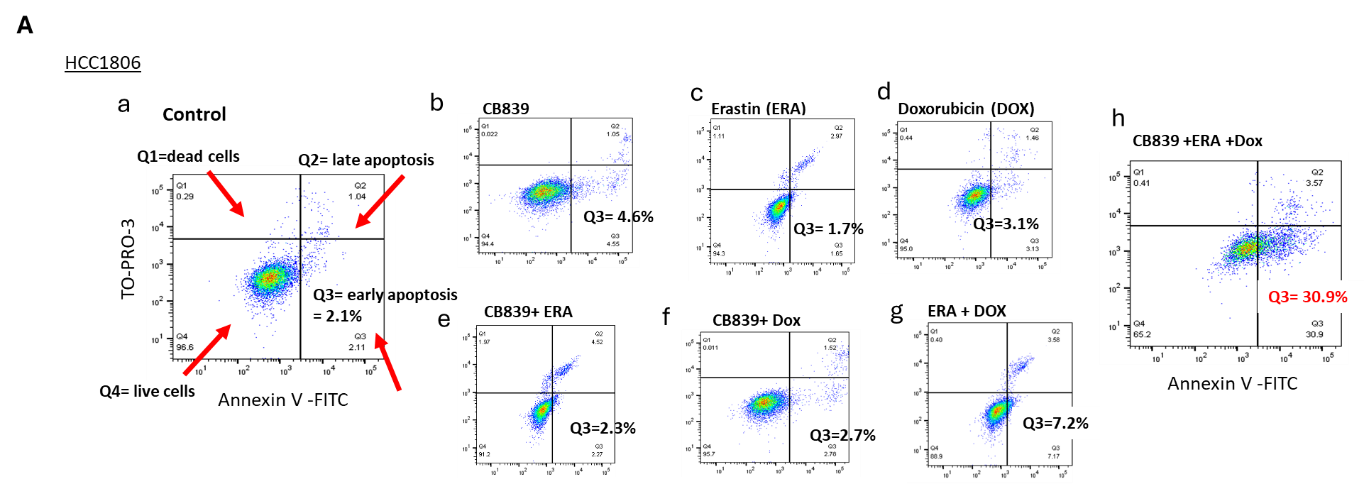
**

**
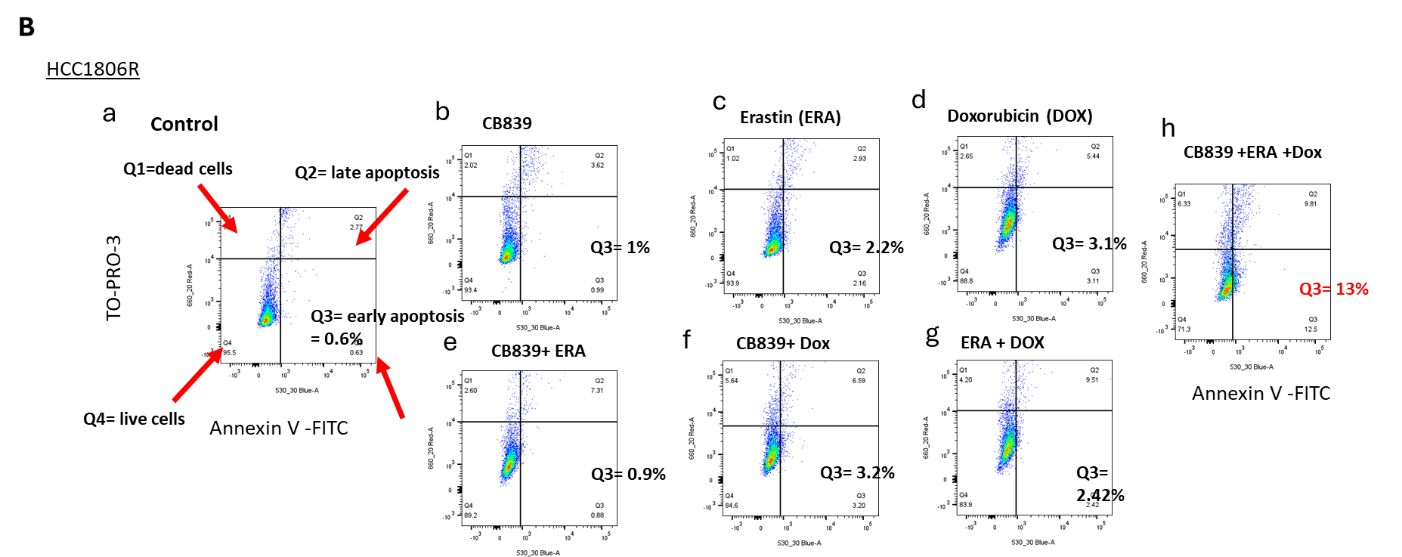
**

**SI Figure 3. FACS based estimation of early apoptosis and dead cell by Annexin V-FITC and TO-PORO-3 respectively, in response to CB839, ERA, DOX and their combinations, respectively.** Fraction of cells undergoing apoptosis (high Annexin V-FITC but low TO-PORO-3 signal) are in the quarter-3 (Q3). Representative FACS results from HCC1806 (**A**) and HCC1806R (**B**).

**
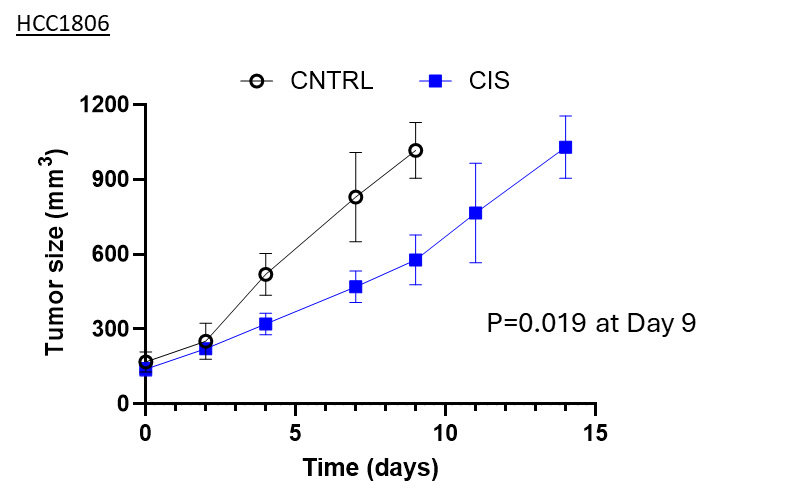
**

**SI Figure 4. CIS treatment induced growth delay in parent (HCC1806) tumors.**

**SI Figure 5. Body weight of mice during treatment described in Figure 6F.** Body weights measured after treatments had no statistical significance compared to those at baseline.

**References**

1. Kagan VE, Mao G, Qu F, Angeli JP, Doll S, Croix CS*, et al.* Oxidized arachidonic and adrenic PEs navigate cells to ferroptosis. Nat Chem Biol **2017**;13:81-90

2. Gambardella G, Viscido G, Tumaini B, Isacchi A, Bosotti R, di Bernardo D. A single-cell analysis of breast cancer cell lines to study tumour heterogeneity and drug response. Nature communications **2022**;13:1714

3. Sanchez-Vega F, Mina M, Armenia J, Chatila WK, Luna A, La KC*, et al.* Oncogenic Signaling Pathways in The Cancer Genome Atlas. Cell **2018**;173:321-37 e10

4. Yoo HC, Yu YC, Sung Y, Han JM. Glutamine reliance in cell metabolism. Experimental & molecular medicine **2020**;52:1496-516
